# Calcium Channel Blocker Overdose Treated with Calcium Resulting in Pancreatitis: A Case Report

**DOI:** 10.7759/cureus.4493

**Published:** 2019-04-18

**Authors:** Salman Khan, Kim J Norville, Imran Khan, Faraz Siddiqui, Apurwa Karki

**Affiliations:** 1 Internal Medicine, Guthrie Clinic/Robert Packer Hospital, Sayre, USA; 2 Internal Medicine - Critical Care, Guthrie Clinic/Robert Packer Hospital, Sayre, USA; 3 Internal Medicine, North Shore University Hospital, Hempstead, USA

**Keywords:** pancreatitis, hypercalcemia, calcium channel blocker, overdose

## Abstract

Calcium channel blockers (CCBs) are a mainstay for the treatment of hypertension. Here we report a case of a male who after intentionally ingesting amlodipine presented with overdose symptomology. His QTc (corrected QT) was 525 ms (millisecond) on admission, he was treated with calcium intravenous infusion and subsequently his QTc narrowed to 393 ms, but he also developed iatrogenic pancreatitis. His serum calcium levels were not checked during the infusion. He was treated with supportive care, which led to the normalization of serum calcium levels and a favorable outcome. Further studies are required regarding how frequently calcium levels should be checked during infusions.

## Introduction

Hypercalcemia is a relatively rare condition with up to 80% of cases considered to be from primary hyperparathyroidism and malignancy, and limited literature is found on hypercalcemia as a complication of intravenous calcium infusion [1]. This case discusses hypercalcemia-induced pancreatitis after infusion of calcium for calcium channel blocker overdose. Supportive care and aggressive hydration corrected the metabolic abnormalities and the patient survived.

## Case presentation

A 57-year-old male was evaluated in the emergency department for lightheadedness. Approximately 12 hours prior to presentation, he intentionally ingested 30 tablets of amlodipine 10 mg with suicidal intent, and afterwards he took a nap. When he woke up, he was unable to move his legs and felt lightheaded. He had been on amlodipine for three years as a treatment for hypertension. His past medical history was significant for chronic alcoholism and human immunodeficiency virus (HIV) apart from hypertension. On arrival, the patient was alert but reported feeling weakness all throughout his body and lightheadedness. On examination, he was found to be bradycardic with a heart rate of 50 beats per minute and he had hypotension with a systolic blood pressure of 70 mm Hg. He was administered 2 liters of intravenous 0.9% saline. His laboratory investigation was remarkable for potassium of 3.2 mmol/L (reference range 3.5-5.1), bicarbonate of 19 mmol/L (reference range 22-30), creatinine of 5.3 mg/DL (reference range 0.82-1.5), and calcium of 8.2 mg/DL (reference range 8.3 to 10.1).

**Figure 1:**
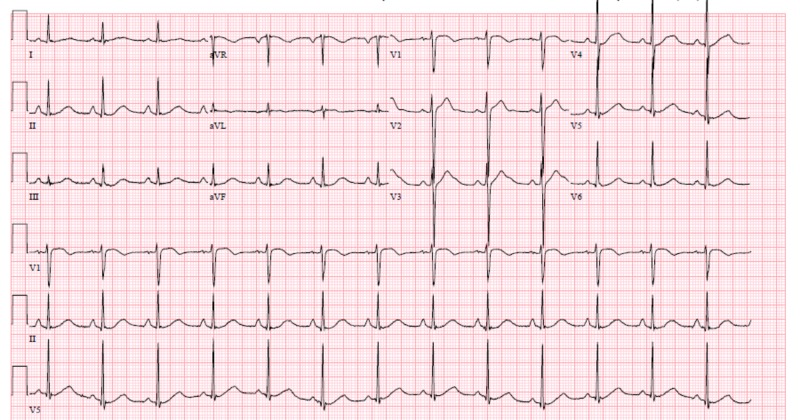
Electrocardiogram (EKG) with QTC of 525 ms

The patient was administered two more liters of 0.9% normal saline as a bolus, and after consultation with the regional Poison Control Center, a recommendation to administer 20 grams of calcium gluconate in dextrose solution was made. The initial QTC on electrocardiogram (EKG) was 525 (Figure 1). The initial EKG showed normal sinus rhythm with prolonged QT interval with U waves. The patient was admitted to the medical intensive care unit (ICU) for further treatment.

**Figure 2:**
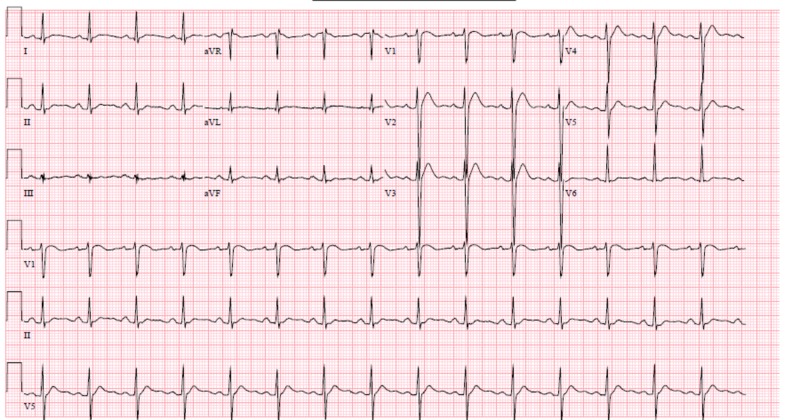
Electrocardiogram (EKG) with QTC 393 ms

**Figure 3:**
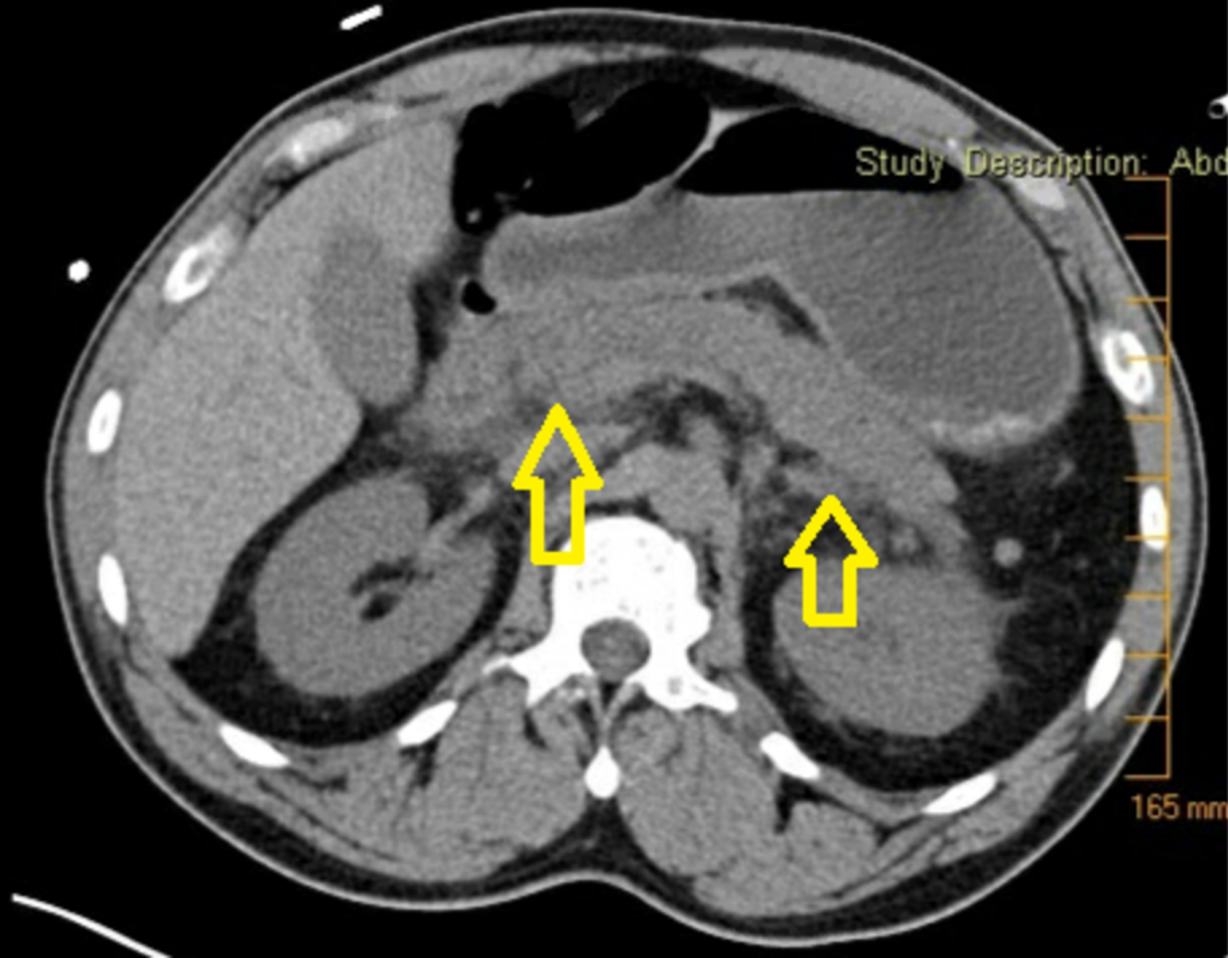
Computed tomography of the abdomen with arrows pointing towards the inflammation seen in pancreatitis

Intravenous infusion of calcium chloride 20 grams in dextrose 5% was administered at the rate of 100 ml/hour. Basic metabolic profile (BMP) drawn prior to the calcium chloride infusion showed potassium 2.7 mmol/L, bicarbonate 17 mmol/L, creatinine 4.7 mg/DL, and calcium 9.3 mg/DL. Approximately six hours after the infusion of calcium chloride was started, the BMP was checked and showed potassium of 3.6 mmol/L as it was supplemented, bicarbonate of 18 mmol/L, creatinine of 2.7 mg/DL, and calcium of 22.7 mg/DL. At that point, the calcium chloride infusion was stopped. EKG at that time showed QTC 393 ms (Figure 2). The serum calcium level was checked five hours later and showed calcium of 19.4 mg/DL. His urine output over the last five hours was 400 to 425 ml an hour. Three hours later, the patient started complaining of severe central abdominal pain. A computer tomography (CT) scan of the abdomen without contrast was obtained and showed significant peripancreatic stranding extending within the anterior pararenal space and tracking down along the right psoas muscle and left psoas muscle into the pelvis. These signs were consistent with acute pancreatitis (Figure 3). Treatment was started with intravenous isotonic solution, and this improved his blood pressure and pain management was achieved with as-needed opiate. Twenty-four hours after arrival into the hospital, his potassium was 4.0 mmol/L, creatinine was 1.3 mg/DL, and calcium was 11.3 mg/DL.

The patient's clinical course gradually improved and he no longer required ICU monitoring and was transferred to the floor. Inpatient psychiatry consultation was placed and he was recommended for admission to the Behavioral Science unit. On the day of discharge, his potassium level was 4.0 mmol/L, creatinine was 1.0 mg/DL, and calcium was 9.5 mg/DL.

## Discussion

Calcium channel blockers (CCBs) are often prescribed for essential hypertension. The overuse of CCBs can lead to arterial vasodilation resulting in hypotension and bradycardia [2]. Patients may also present with altered sensorium and hyperglycemia due to reduced insulin secretion [3].

The management of CCB overdose is to optimize intravascular volume and restore cardiac output. If the patient develops hypotension, the addition of inotropes may be warranted. Intravenous calcium, insulin or glucagon can be utilized as well [4]. Another modality is sodium bicarbonate, which can be especially useful in the presence of metabolic acidosis and, if used, may improve the hemodynamic status [5].

In an observational study conducted over one year, 139 patients were studied with calcium channel blocker overdose. Calcium was administered to 23 patients and it demonstrated improvements in hemodynamics but no specific dose was established [6]. Another study investigated dosage of calcium administration. The authors utilized a loading dose of 0.6 ml kg-1 of 10% calcium gluconate and infusion of 0.6-1.6 ml/kg/hour titrated to hemodynamic parameters and a serum ionized calcium up to two times the upper limit of the reference range [7]. In our case, after consultation with the regional poison control, we opted to use 20 grams of calcium gluconate.

As mentioned above, infusion of calcium via intravenous therapy is beneficial for hemodynamic stability in CCB overdose. However, no specific dose has been established. High normal ranges to twice the normal range of calcium are advised [3]. One case report demonstrated a calcium level of 32.3 mg/DL that induced iatrogenic pancreatitis leading to anuric kidney injury, which required continuous renal replacement therapy but the patient ultimately succumbed to his injuries [8]. In our case, our highest level of calcium was 22.7 g/dl, which induced pancreatitis. If calcium levels had been measured more frequently, the calcium dose might have been adjusted earlier, possibly reducing the risk of an onset of pancreatitis in our patient. The first calcium level was not checked until six hours after transfusion was initiated. We suggest frequent measurements of serum calcium and goal‐directed tapering of the infusion rate as do the authors Van Veggel et al. [9]. There are no guidelines available for the management of CCB overdose with intravenous calcium or monitoring for hypercalcemia; however, utilizing clinical judgement and the patient’s condition can be the driving factor.

## Conclusions

The treatment of calcium channel blocker overdose can be challenging and unique. Overdose of calcium channel blockers can result in myocardial depression along with arterial vasodilation. Vasodilatory shock can play a part in their toxicity and the use of vasopressors might be needed. Intravenous calcium administration can have an effect on contractility, and careful monitoring is suggested. In our case the use of intravenous calcium therapy was utilized as suggested by poison control, but given the large dose, frequent monitoring during the infusion was needed to prevent pancreatitis. This was treated with aggressive intravenous normal saline therapy.

## References

[1] Lafferty FW (1991). Differential diagnosis of hypercalcemia. J Bone Miner.

[2] DeWitt CR, Waksman JC (2004). Pharmacology, pathophysiology and management of calcium channel blocker and beta blocker toxicity. Toxicol Rev.

[3] Graudins A, Lee HM, Druda D (2016). Calcium channel antagonist and beta-blocker overdose: antidotes and adjunct therapies. Br J Clin Pharmacol.

[4] Salhanick SD, Shannon MW (2003). Management of calcium channel antagonist overdose. Drug Saf.

[5] Kumar S, Thakur D, Gupta RK, Sharma A (2018). Unresponsive shock due to amlodipine overdose: an unexpected cause. J Cardiovasc Thorac Res.

[6] Ramoska EA, Spiller HA, Winter M, Borys D (1993). A one‐year evaluation of calcium channel blocker overdoses: toxicity and treatment. Ann Emerg Med.

[7] Kerns W II (2007). Management of beta‐adrenergic blocker and calcium channel antagonist toxicity. Emerg Med Clin North Am.

[8] Sim MT, Stevenson FT (2008). A fatal case of iatrogenic hypercalcemia after calcium channel blocker overdose. J Med Toxicol.

[9] Van Veggel M, Van der Veen G, Jansen T, Westerman E (2017). A critical note on treatment of a severe diltiazem intoxication: high‐dose calcium and glucagon infusions. Basic Clin Pharmacol Toxicol.

